# Prediction of neoadjuvant chemotherapy response in breast cancer

**DOI:** 10.17179/excli2021-3607

**Published:** 2021-03-10

**Authors:** Maiju Myllys

**Affiliations:** 1 Leibniz Research Centre for Working Environment and Human Factors

## ⁯⁯⁯⁯

***Dear Editor,***

Neoadjuvant chemotherapy (NACT) in breast cancer reduces the size of breast carcinomas to improve operability (Burstein et al., 2019[2]; Ditsch et al., 2019[7]). The response to NACT is critical since a pathologic complete response (pCR) is associated with better long-term survival (Spring et al., 2020[24]). Unfortunately, the response rates to currently used NACT with taxanes and anthracyclines are relatively low with pCR between 15 and 40 % (von Minckwitz et al., 2008[28], 2012[27]; Iwata et al., 2011[18]; Untch et al., 2016[26]; Gianni et al., 2018[11]). Therefore, a classifier that reliably predicts non-response (non-pCR) to taxanes/anthracyclines would be of high relevance, sparing the patients from unnecessary toxicity due to the chemotherapy. Moreover, alternative treatments could be chosen. Unfortunately, currently available expression-based classifiers do not guarantee sufficiently high negative prediction values (NPV) to justify clinical decisions (Chang et al., 2003[6]; Ayers et al., 2004[1]; Hess et al., 2006[17]; Farmer et al., 2009[10]; Hatzis et al., 2011[13]). 

Recently, Edlund and colleagues have established an expression-based classifier for prediction of neoadjuvant chemotherapy response that offers some important advantages (Edlund et al., 2021[8]). Initially, the authors realized that it is difficult to establish a classifier that informs each patient whether they will respond to NACT, which is in agreement with previous studies. Therefore, they constructed a classifier that allows very reliable statements but only for a subset of the patients. In the present publication, Edlund et al., present a 20-gene classifier that identifies non-responders with an unusually high NPV of 0.960 considering all four intrinsic subtypes of breast cancer. In patients with the luminal A subtype, NPV was even as high as 0.986 (Edlund et al., 2021[8]). This classifier was trained in a new prospective multicenter trial with 114 patients and validated in a cohort of 619 independent patients with taxane/anthracycline-based NACT. Prediction of the prognosis of breast cancer based on gene expression has been studied since decades (Schmidt et al.,, 2008[20]). Prognostic and predictive genes comprise marker genes of immune cells (Schmidt et al., 2012[21], 2018[22]; Godoy et al., 2014[12]; Heimes et al., 2017[14][15]; Edlund et al., 2019[7]), proliferation associated genes (Siggelkow et al., 2012[23]), oxidative stress response (Cadenas et al., 2010[3]), metabolism (Stewart et al., 2012[25]; Cadenas et al., 2014[4], 2019[5]; Marchan et al., 2017[19]), and ribosome-related genes (Hellwig et al., 2016[16]). 

The present study of Edlund and colleagues represents an important progress in research on NACT with the limitation that a reliable recommendation of not to treat can only be made for a subgroup of the patients. The novel 20-gene classifier will become clinically relevant as soon as alternative treatments to taxane/anthracycline-based NACT such as inhibitors of PARP, cyclin-dependent kinases 4/6 or immune checkpoints will be introduced into clinical routine. In this case, patients that will not respond to the conventional taxane/anthracycline-based therapy will be reliably identified by the present classifier and hence can be treated with a more efficient alternative. 

## Conflict of interest

The author declares no conflict of interest.
